# An LPA species (18:1 LPA) plays key roles in the self-amplification of spinal LPA production in the peripheral neuropathic pain model

**DOI:** 10.1186/1744-8069-9-29

**Published:** 2013-06-17

**Authors:** Lin Ma, Jun Nagai, Jerold Chun, Hiroshi Ueda

**Affiliations:** 1 Department of Molecular Pharmacology and Neuroscience, Nagasaki University Graduate School of Biomedical Sciences, 1-14 Bunkyo-machi, Nagasaki 852-8521, Japan; 2 Department of Molecular Biology, Dorris Neuroscience Center, The Scripps Research Institute, 10550 North Torrey Pines Road, ICND118, La Jolla, CA 92037, USA

**Keywords:** Lysophosphatidic acid, Neuropathic pain, MALDI-TOFMS, Phos-tag, Cytosolic phospholipase A_2_, Neuron

## Abstract

**Background:**

We previously reported that nerve injury-induced neuropathic pain is initiated by newly produced lysophosphatidic acid (LPA).

**Results:**

In this study, we developed a quantitative mass spectrometry for detecting LPA species by using Phos-tag. Following nerve injury, the levels of 18:1, 16:0 and 18:0 LPA in the spinal dorsal horn significantly increased at 3 h and declined at 6 h. Among them, 18:1 LPA level was the most abundant. In the same preparation, there were significant elevations in the activities of cytosolic phospholipase A_2_ (cPLA_2_) and calcium-independent phospholipase A_2_ (iPLA_2_), key enzymes for LPA synthesis, at 1 h, while there was no significant change in phospholipase A_1_ activity. Pharmacological studies revealed that NMDA and neurokinin 1 receptors, cPLA_2_, iPLA_2_ and microglial activation, as well as LPA_1_ and LPA_3_ receptors were all involved in the nerve injury-induced LPA production, and underlying cPLA_2_ and iPLA_2_ activations. In the cells expressing LPA_1_ or LPA_3_ receptor, the receptor-mediated calcium mobilization was most potent with 18:1 LPA, compared with 16:0 or 18:0 LPA. Moreover, the intrathecal injection of 18:1 LPA, but not 16:0 or 18:0 LPA, caused a spinal LPA production and neuropathic pain-like behavior.

**Conclusion:**

These results suggest that 18:1 LPA is the predominant ligand responsible for LPA_1_ and LPA_3_ receptors-mediated amplification of LPA production through microglial activation.

## Background

Lysophosphatidic acid (LPA) is a lysophospholipid with a structure comprising a glycerol backbone, a free phosphate group, and a single fatty acyl chain. Usually, LPA consists of several molecular species with different acyl chains varing in the *sn*-1 or *sn*-2 position, the length and degree of saturation [1,2]. LPA is an important biological signaling molecule, which is generated in several biological fluids such as serum, saliva and follicular fluid [3-5], and in most tissues such as brain, spinal cord and lung [6,7]. Its landmark roles in various physiological and pathophysiological conditions, including wound healing, lung fibrosis, cancer, reproduction, and hair growth, have been well-documented [8-12]. These biological functions have been identified to link with specific G-protein coupled receptors named LPA_1-6_[13,14].

In recent decade, LPA has gained special attention because of its emerging role as an important risk factor in chronic neuropathic pain [15-18]. In these studies, we found the roles of LPA_1_ signaling for the initiation of neuropathic pain and its underlying mechanisms, such as demyelination [19-24]. Among these reports, it should be noted that the single intrathecal (i.t.) injection of LPA mimicked the behavioral changes, demyelination and several biochemical changes caused by nerve injury [20,23,25]. This unique approach to study neuropathic pain and its mechanisms was further confirmed by other groups [26-28]. Emerging findings were observed with the studies of biosynthesis of LPA in the spinal cord or dorsal root in the neuropathic pain model. The LPA production, measured by biological assay, reached a maximum at 3 h after the injury [6], being consistent with the pharmacological study that the blockade of neuropathic pain was critically observed when LPA_1_ receptor antagonist was treated within 2 – 4 h, but not at 6 h [21]. Similar LPA production was also found with i.t. LPA treatment [29], suggesting that LPA itself plays important roles in the amplification of LPA biosynthesis. Interestingly, LPA-induced amplification of LPA production was abolished in *Lpar3*-deficient (*Lpar3*^*-/-*^) mice [29], indicating the involvement of LPA_3_ receptor. Moreover, it should be noted that microglial activation was also involved in this LPA production [30].

On the other hand, we successfully identified the species of lysophosphatidylcholine (LPC), the precursor of LPA, in the spinal dorsal horn after the nerve injury [31]. In this experiment, the LPC species were simultaneously determined by use of nanostructure-assisted laser desorption/ionization time-of-flight mass spectrometry system, which removed the need to add chemical matrices for the analysis of small molecules, and enabled to minimize their background noises [32]. However, it was difficult to quantitate or even detect any species of LPA by this system, because of poor ionization due to its acidic nature and multiple signals derived from free and salt forms. Recently, this problem was solved by derivatization of LPA molecules using a phosphate-capture molecule, Phos-tag [33], followed by matrix-assisted laser desorption/ionization time-of-flight mass spectrometry (MALDI-TOFMS) system [7].

In the present study, we attempted to measure the LPA production in the spinal dorsal horn following sciatic nerve injury by use of this LPA derivatization methodology, and identified the key species of LPA in charge of amplification of LPA production.

## Results

### Determination of LPA by MALDI-TOFMS using Phos-tag

**Figure 1:**
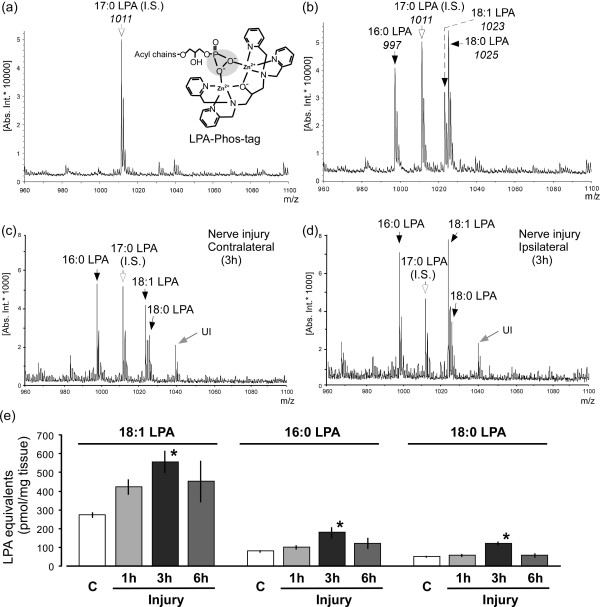
**Biosynthesis of LPA following nerve injury by MALDI-TOFMS using Phos-tag.** (**a** and **b**) Representative charts of mass spectra with internal standard 17:0 LPA *(panel* ***a****)*, and mixed standard reagents 16:0, 18:0 and 18:1 LPA as well as internal control *(panel* ***b****)* at the level of 0.2 nmol, following the use of Phos-tag. (**c**  and **d**) Mass spectra of LPA extracts from the contralateral *(panel* ***c****)* and ipsilateral *(panel* ***d****)* dorsal half of spinal cord at 3 h after nerve injury. “UI” represents the unidentified compound. (**e**) Levels of produced LPA species (18:1, 16:0, 18:0 LPA) in spinal cord preparations of control or injured mice. The capital letter “C” represents the control group (naive mice). Data represent means ± SEM from experiments using 3-8 mice. *p < 0.05, versus with the control group.

To detect LPA species, in this study, we developed a quantitative MALDI-TOFMS method by use of a phosphate-capture molecule, Phos-tag, according to previous reports [7,33,34]. Figure 1a and b present the MALDI-TOF mass spectrum of internal standard 17:0 LPA, as well as mixture of authentic standard reagents 16:0, 18:0 and 18:1 LPA with the internal standard at the level of 0.2 nmol, respectively. Distinctive peaks were detected at *m/z* 1011, 997, 1023 and 1025, which correspond to 17:0 LPA-Phos-tag, 16:0 LPA-Phos-tag, 18:1 LPA-Phos-tag and 18:0 LPA-Phos-tag, respectively (Figure 1a and b).

In order to quantify the levels of these LPA species, we applied each standard LPA (16:0, 18:0 and 18:1 LPA) at 0, 0.1, 0.2, 0.5, 1.0 and 2.0 nmol with 17:0 LPA at 0.2 nmol into MALDI-TOFMS system. According to the ratios of ion-peak intensities with each standard LPA to that with 17:0 LPA, the concentration-related linear equations were established after subtracting the basal background. They were defined as y = 0.9928x (R^2^ = 0.9975; x: concentration ratio of 16:0 to 17:0 LPA, y: intensity ratio of 16:0 to 17:0 LPA), y = 1.4122x (R^2^ = 0.9923; x: concentration ratio of 18:0 to 17:0 LPA, y: intensity ratio of 18:0 to 17:0 LPA) and y = 0.3956x (R^2^ = 0.9971; x: concentration ratio of 18:1 to 17:0 LPA, y: intensity ratio of 18:1 to 17:0 LPA), responding to 16:0, 18:0 and 18:1 LPA, respectively. In subsequent studies, LPA equivalents in the extracts from solid tissue were estimated using these equations based on linear LPA concentration-dependent responses.

### Time-related elevation of nerve injury-induced LPA production

We analyzed LPA levels in the spinal dorsal horn after nerve injury by the MALDI-TOFMS system. As shown in the representative mass spectra of contralateral (control side) and ipsilateral (injured side) spinal dorsal horn at 3 h after nerve injury (Figure 1c and d), marked increases of the ion-signal were observed at *m/z* 997, 1023 and 1025 in the ipsilateral spinal cord, corresponding to 16:0, 18:1 and 18:0 LPA, respectively.

LPA equivalents were calculated based on the linear equations of each LPA. As shown in Figure 1e, after nerve injury, the 18:1 LPA level peaked at 3 h, followed by the slight decline at 6 h. Quite similar changes were also observed in the 16:0 and 18:0 LPA production (Figure 1e).

### Blockade of nerve injury-induced LPA production

**Figure 2:**
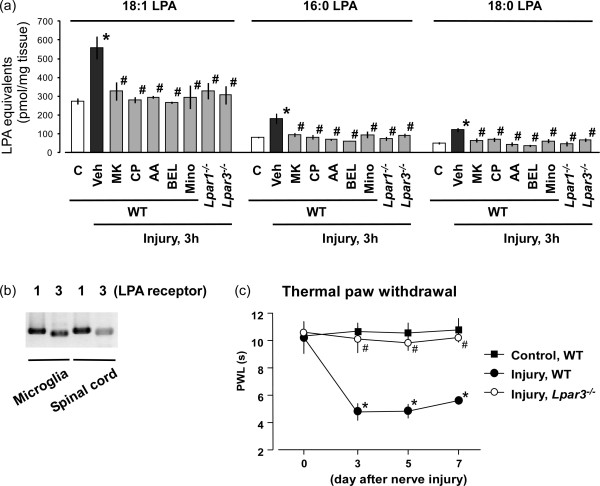
**Blockade of nerve injury-induced LPA production.** (**a**) Following pre-treatments of vehicle, MK-801, CP-99994, AACOCF3, BEL (each 10 nmol, i.t.) and minocycline (30 mg/ml, i.p.) before nerve injury, the ipsilateral spinal dorsal horn of each mouse was isolated at 3 h after injury. Levels of LPA species (18:1, 16:0, 18:0 LPA) were measured using such spinal cord preparations by MALDI-TOFMS. Besides, *Lpar1*- and *Lpar3*-deficient mice were also used to evaluate the LPA production at 3 h after injury. The capital letter “C” represents the control group (naive mice). The “veh”, “MK”, “CP”, “AA” and “mino” represent vehicle, MK-801, CP-99994, AACOCF3 and minocycline, respectively. (**b**) RT-PCR experiment was carried out to evaluate the expressions of LPA_1_ and LPA_3_ receptors in cultured mouse microglia and mouse spinal dorsal horn. (**c**) Thermal paw withdrawal test was performed at time-course points after nerve injury using wild-type or *Lpar3*-deficient mice. Results represent the threshold of latency (s) to thermal stimulus. “WT” represents the wide-type mice. Data represent means ± SEM from experiments using 3-8 mice. *p < 0.05, versus with the control group; #*p* < 0.05, versus with the vehicle-injury or injury-WT group.

Nerve injury causes the release of pain transmitters such as glutamate (Glu) and substance P (SP) from primary afferent fibers, and they activate NMDA and neurokinin 1 (NK1) receptors at the dorsal horn, respectively [18]. Although their roles in neuropathic pain are well known, the extent of their contribution to LPA production remains unclear. Here, we injected MK-801 or CP-99994 (10 nmol, i.t.), the antagonists of NMDA or NK1 receptor, respectively [35], at 30 min prior to nerve injury, and found that both of them significantly blocked nerve injury-induced LPA production with three species at 3 h (Figure 2a).

Since cytosolic phospholipase A_2_ (cPLA_2_) and calcium-independent phospholipase A_2_ (iPLA_2_) catalyze phosphatidylcholine (PC) conversion to LPC [35-37], in this study, we pre-treated arachidonyl trifluoromethyl ketone (AACOCF3; 10 nmol, i.t.), a mixed inhibitor of cPLA_2_ and iPLA_2_[38,39], or bromoenol lactone (BEL; 10 nmol, i.t.), a specific iPLA_2_ inhibitor [38], at 30 min before nerve injury. As shown in Figure 2a, both inhibitors abolished the nerve injury-induced LPA production (Figure 2a).

Similarly, minocycline [30 mg/kg, intraperitoneal (i.p.)], a microglial activation antagonist [30], completely blocked injury-induced LPA production (Figure 2a), when it was pre-treated twice at 1 day and 30 min before injury. In addition, reverse transcription polymerase chain reaction (RT-PCR) experiments showed that both LPA_1_ and LPA_3_ receptors are expressed in cultured mouse microglia and mouse spinal dorsal horn (Figure 2b). Nociceptive tests demonstrated that *Lpar3*^*-/-*^ mice completely abolished the nerve injury-induced thermal hyperalgesia (Figure 2c). Considering the fact that *Lpar1*-deficient (*Lpar1*^*-/-*^) mice also showed no neuropathic pain behavior and underlying mechanisms [20,29], we attempted to see whether the injury-induced LPA production was affected in *Lpar1*^*-/-*^ mice, as well as in *Lpar3*^*-/-*^ mice. As shown in Figure 2a, the LPA levels at 3 h after injury were also abolished in mice deficient of either gene, compared with wild-type mice.

### Blockades of elevated nerve injury-induced cPLA_2_ and iPLA_2_ activities

**Figure 3:**
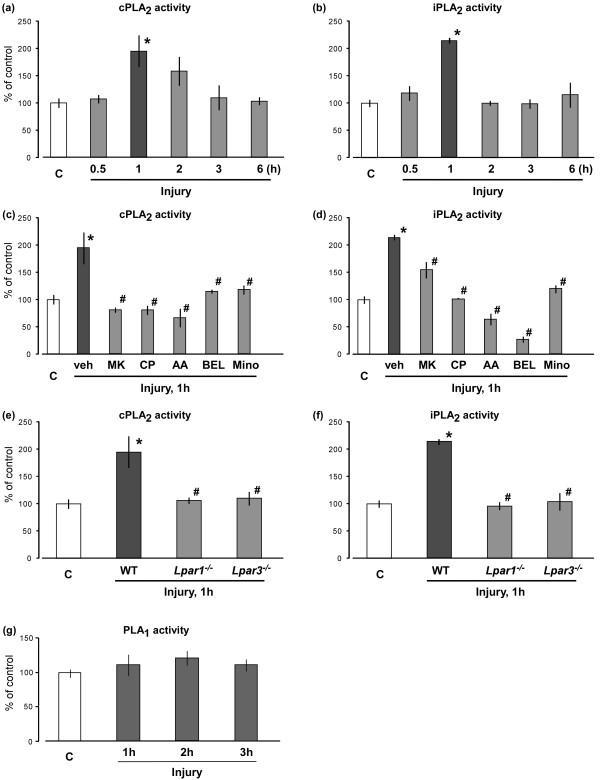
**Blockade of nerve injury-induced cPLA**_**2 **_**and iPLA**_**2 **_**activations.** (**a** and **b**) Activation of spinal cPLA_2_* (panel* ***a****)* and iPLA_2_* (panel* ***b****)* were detected by cPLA_2_ and iPLA_2_ activity assays at defined time points after nerve injury. The capital letter “C” represents the control group (naive mice). (**c** and **d**) After pre-treatments of vehicle, MK-801, CP-99994, AACOCF3, BEL (each 10 nmol, i.t.) and minocycline (30 mg/ml, i.p.) before nerve injury, the activities of spinal cPLA2 *(panel* ***c****)* and iPLA2 *(panel* ***d****)* at 1 h after injury were evaluated. The “veh”, “MK”, “CP”, “AA” and “mino” represent vehicle, MK-801, CP-99994, AACOCF3 and minocycline, respectively. (**e** and **f**) Activities of cPLA_2_*(panel* ***e****)* and iPLA_2_* (panel* ***f****)* were measured at 1 h after injury using the *Lpar1*- and *Lpar3*-deficient mice. “WT” represents the wide-type mice. (**g**) PLA_1_ activity in the spinal dorsal horn was measured by PLA_1_ activity assay at time-course points after nerve injury. Data represent means ± SEM from experiments using 3-6 mice. *p < 0.05, versus with the control group; #*p* < 0.05, versus with the vehicle/WT-injury group.

In this study, the nerve injury-induced activations of cPLA_2_ and iPLA_2_ in the spinal dorsal horn were evaluated by cPLA_2_ and iPLA_2_ activity assays. The enzyme activity of cPLA_2_ was maximal at 1 h, and slowly declined to the control level at 3 h (Figure 3a). Although the maximal activity of iPLA_2_ was also observed at 1 h, the decline was rapid (Figure 3b). The enhanced cPLA_2_ activity was abolished by MK-801, CP-99994, AACOCF3 or BEL (each 10 nmol, i.t.), all of which had been pretreated 30 min before the nerve injury. The pretreatments of minocycline (30 mg/kg, i.p.) 1 day and 30 min before the injury also abolished the enhanced cPLA_2_ activity (Figure 3c). All these inhibitors also significantly inhibited the enhanced iPLA_2_ activity, though their sensitivities against iPLA_2_ were varied among inhibitors and different from those against cPLA_2_ (Figure 3d). Among them, it was noted that MK-801-induced inhibition of iPLA_2_ was partial, while BEL inhibited the activity to the level lower than the uninjured control. Similar complete blockade was observed in *Lpar1*^*-/-*^ or *Lpar3*^*-/-*^ mice (Figure 3e and f). However, as shown in Figure 3g, there was no significant change in phospholipase A_1_ (PLA_1_) activity after nerve injury.

### Cell-type identification of activated cPLA_2_ in spinal cord

**Figure 4:**
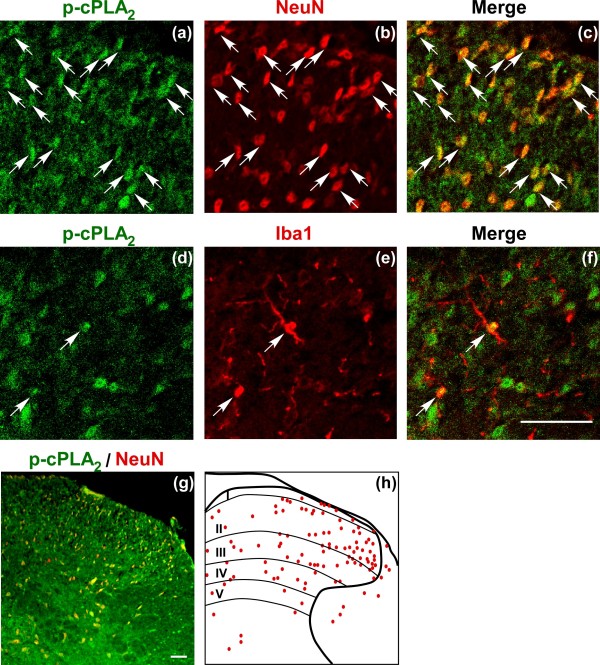
**Cell-type identification of activated cPLA**_**2 **_ **in spinal cord.** (**a-f**) Immunohistochemical double labeling of p-cPLA_2_ (green) and NeuN (red) as well as p-cPLA_2_ (green) and Iba1 (red) in the spinal cord at 1 h after nerve injury. White arrows represent NeuN- or Iba1-co-localized p-cPLA_2_ signals. (**g**) Low magnification of double immunostaining with p-cPLA_2_ (green) and NeuN (red) in the spinal cord at 1 h after injury. (**h**) Diagram of Figure 4g. Red dots represent NeuN co-localized p-cPLA_2_ signals. Scale bar (including high and low magnifications), 50 μm.

In order to identify the cell-type expressing activated (phosphorylated) cPLA_2_ (p-cPLA_2_) in the spinal cord, we performed double immunostaining of p-cPLA_2_ with antibodies against two kinds of cell-specific markers: neuronal nuclei (NeuN; neuron) and ionized calcium-binding adaptor molecule 1 (Iba1; microglia). At 1 h after nerve injury, most of p-cPLA_2_ signals co-localized with NeuN-positive neurons (Figure 4a-c), whose distributions were diffused expressed throughout laminae I-IV layers of the spinal dorsal horn (Figure 4g and h). On the other hand, a few signals were also observed in Iba1-positive microglia (Figure 4d-f). However, there was no p-cPLA_2_ signal in glial fibrillary acidic protein (astrocyte marker)-positive cells (data were not shown).

### Pharmacological blockade of nerve injury-induced cPLA_2_ phosphorylation

**Figure 5:**
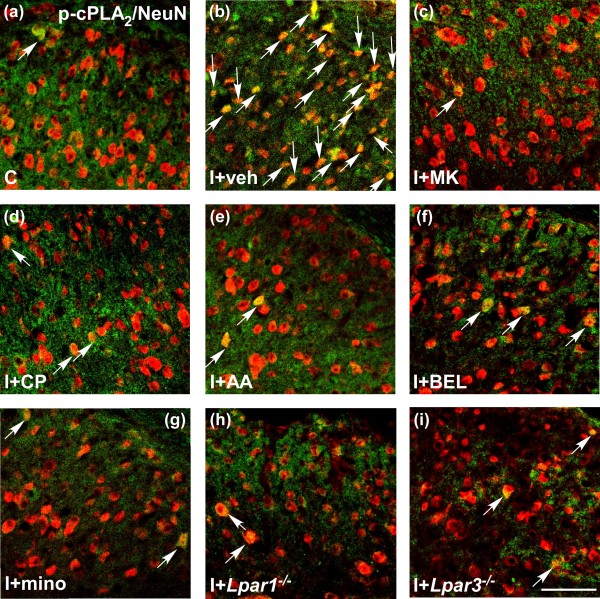
**Blockade of p-cPLA**_**2 **_ **signals in spinal neurons after nerve injury.** (**a-i**) Vehicle, MK-801, CP-99994, AACOCF3, BEL (each 10 nmol, i.t.) and minocycline (30 mg/ml, i.p.) were pre-treated before nerve injury, and the spinal cord were collected at 1 h after injury, which were used for the double immunohistochemistry with p-cPLA_2_ (green) and NeuN (red). Control as well as *Lpar1*- and *Lpar3*-deficient mice were also used in this experiment. White arrows represent NeuN-co-localized p-cPLA_2_ signals. The capital letter “C” and “I” represent control and nerve injury, respectively. The “veh”, “MK”, “CP”, “AA” and “mino” represent vehicle, MK-801, CP-99994, AACOCF3 and minocycline, respectively. Scale bar, 50 μm.

Double immunostaining with antibodies against p-cPLA_2_ and NeuN was performed in the spinal cord of control and pharmacological antagonists-pretreated injured mice. In control group (naive mice), p-cPLA_2_ showed a low expression in neurons. However, nerve injury induced a significant increase in neuronal p-cPLA_2_ signals at 1 h after injury, and the increase was blocked by various inhibitors, such as MK-801, CP-99994, AACOCF3, BEL and minocycline, as well as in *Lpar1*^*-/-*^ and *Lpar3*^*-/-*^ mice (Figure 5). These findings were in good accordance with those observed in experiments of LPA detection and cPLA_2_ and iPLA_2_ activity assays.

### Comparison of LPA_1_ or LPA_3_ agonist activities by different species of LPA

**Figure 6:**
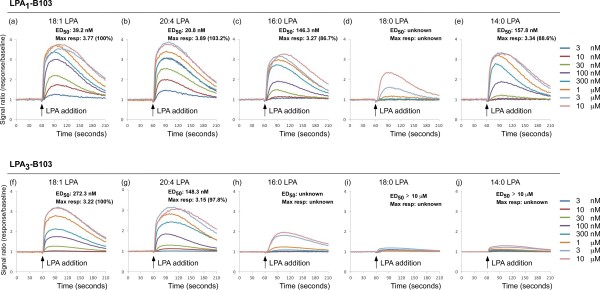
**LPA**_**1 **_**or LPA**_**3 **_**receptor-mediated calcium mobilization by exogenous LPA species.** After the addition of 18:1 *(panel* ***a**** and* ***f****)*, 20:4 *(panel* ***b**** and* ***g***), 16:0 *(panel* ***c**** and* ***h****)*, 18:0 *(panel* ***d**** and* ***i****)* or 14:0 *(panel* ***e**** and* ***j****)* LPA with defined concentrations (3, 10, 30, 100, 300 nM and 1, 3, 10 μM) into the B103 cells expressing LPA_1_ or LPA_3_ receptor, calcium mobilization assay was immediately performed. “ED_50_” and “Max resp” represent the half-maximal effective dose and maximal response, respectively.

In order to evaluate the agonist potency of each LPA species, calcium mobilization assay was performed using B103 cells expressing LPA_1_ or LPA_3_ receptor (LPA_1_-B103 or LPA_3_- B103 cells), because both LPA_1_ and LPA_3_ receptors enable to induce calcium release from intracellular stores by activating G_q/11_-PLCβ-IP3 pathway. As seen in Figure 6a, when 18:1 LPA at 1-10 μM was added into the LPA_1_-B103 cells, a transient increase of cytosolic calcium was immediately observed, with a maximum at around 30 s after addition, followed by a gradual decline. Obvious concentration-dependent calcium mobilizations were observed in the range of 3 to 300 nM of 18:1 LPA. The half-maximal effective dose (ED_50_) for 18:1 LPA-induced calcium mobilization in LPA_1_-B103 cells was calculated as 39.2 nM (Figure 6a). As shown in Figure 6a-e, similar ED_50_ (20.8 nM) was also observed with 20:4 LPA, but little higher values were with 16:0 and 14:0 LPA (146.3 and 167.8 nM, respectively). When the maximal effect of 18:1 LPA was evaluated as 100%, these values of 20:4, 16:0 and 14:0 LPA were 103.2, 86.7 and 88.6%, respectively. However, as no evident maximal effect was obtained with 18:0 LPA in the range of concentrations we used, its ED_50_ value was not determined. Similar results were also observed in the cases with 18:1 and 20:4 LPA on LPA_3_-B103 cells (Figure 6f and g). The values of ED_50_ were 272.3 and 148.3 nM, respectively, and maximal responses were 100% and 97.8%, respectively. Compared with these two species of LPA, the calcium mobilization by 16:0, 18:0 and 14:0 LPA was so weak that their values of ED_50_ and maximal responses were not determined (Figure 6h-j).

### Measurement of amplified LPA production by exogenous LPA injection

**Figure 7:**
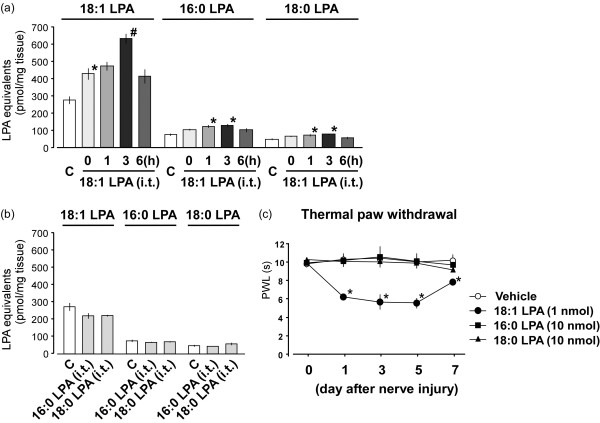
**Amplification of LPA production by injection of LPA species.** (**a**) Levels of LPA species (18:1, 16:0 and 18:0 LPA) in the spinal dorsal horn were measured by MALDI-TOFMS system at time-course points after i.t. 18:1 LPA injection with 1 nmol. (**b**) Measurements of LPA species were performed at 3 h after 16:0 or 18:0 LPA injection with 10 nmol*.* The capital letter “C” represents the control group (naive mice). (**c**) After i.t. injection of 18:1 LPA (1 nmol), 16:0 LPA (10 nmol) or 18:0 LPA (10 nmol), the pain behavior was evaluated by thermal paw withdrawal test. Results represent the threshold of latency (s) to thermal stimulus. Data represent means ± SEM from experiments using 3-7 mice. *p < 0.05, versus with the control/vehicle group; #*p* < 0.05, versus with the 0 h-LPA group.

We previously demonstrated that i.t. LPA injection enabled to feed-forward amplify LPA [29]. In order to identify the key species of LPA molecule in charge of LPA amplification, we i.t. injected three species of LPA (18:1, 16:0 and 18:0 LPA), which were produced by nerve injury, and evaluated amplified LPA production by use of MALDI-TOFMS system. As shown in Figure 7a, after 18:1 LPA injection at 1 nmol, 18:1 LPA itself was newly produced, and the level immediately increased (indicated as 0 h). The elevation may be attributed to the sum of the basal and injected 18:1 LPA. Subsequently, the progressive increase in the level of 18:1 LPA was observed at 1 h, reached a maximum at 3 h, and slightly declined at 6 h. Besides 18:1 LPA, 16:0 and 18:0 LPA were also newly produced after 18:1 LPA injection. The levels of these species of LPA were significantly increased at 1 and 3 h, and slightly decreased at 6 h (Figure 7a). On the other hand, the i.t. administration of 16:0 or 18:0 LPA at a high dose of 10 nmol failed to produce any LPA production at 3 h (Figure 7b). Similarly, in the nociceptive behavior experiments, 18:1 LPA injection with 1 nmol induced neuropathic pain-like thermal hyperalgesia, but 16:0 or 18:0 LPA with 10 nmol did not (Figure 7c).

## Discussion

This study demonstrates three major findings for the first time. First, LPA with three species (18:1, 16:0 and 18:0 LPA) were produced after nerve injury with the use of MALDI-TOFMS system. Second, p-cPLA_2_-expressed neuron was the potent cell to release LPA through LPA_1_ and LPA_3_ receptors-mediated microglial activation. Third, 18:1 LPA was a key ligand to induce amplification of LPA production in the peripheral neuropathic pain model.

The present study successfully detected and quantified several species of LPA molecules after nerve injury through MALDI-TOFMS system with the use of Phos-tag, a zinc complex that specifically binds to a phosphate group [7,33,34]. This MS analysis using Phos-tag significantly decreased the detection limit of LPA compared with previous methods without Phos-tag [7,34]. Moreover, this technique improved our previous biological titration method, because previous one depended solely on the activity of LPA_1_[6,29,35], but not LPA_3_ receptor, which was the important determinant of LPA synthesis [29]. Here, we found that three species of LPA, including 18:1, 16:0 and 18:0 LPA, were maximally produced in the ipsilateral side of spinal dorsal horn, but not the contralateral side, at 3 h after injury, followed by a decline at 6 h. The time-course changes of LPA production was in agreement with previous LPA measurements [6]. This data firstly provided the chemical identification of produced LPA after nerve injury, which was consistent with the molecular species composition of produced LPC (18:1, 16:0 and 18:0 LPC) [31]. Moreover, the quantitative levels of peak 18:1 LPA was approximately three- or four-folds than that of 16:0 or 18:0 LPA, respectively, suggesting that 18:1 LPA was the predominant molecular specie of spinal LPA production after nerve injury. However, there was an interesting finding that in previous LPC measurements, the amount of produced 18:1 LPC was equivalent to or slightly lower than that of 16:0 and 18:0 LPC at 75 min after nerve injury [31]. The difference may be attributed to the biochemical fact that autotaxin, an enzyme to catalyze LPC conversion to LPA, has the preferential affinity to 18:1 LPC than to 16:0 and 18:0 LPC [40].

In this study, pharmacological antagonist of NMDA or NK1 receptor (MK-801 or CP-99994, respectively) completely blocked the nerve injury-induced LPA production. Considering the fact that combination treatments of SP and Glu (or NMDA), but not single treatment, *in vitro* induced LPA production in spinal cord slices [35], we believed that simultaneous intense stimulation of NMDA and NK1 receptors was essential for new LPA biosynthesis in the spinal cord. This proposition was supported by the *ex vivo* study that capsaicin-induced LPA production in spinal cord slices was completely blocked by MK-801 or CP-99994 [35].

Additionally, both cPLA_2_ and iPLA_2_ were maximally activated at 1 h after injury, being consistent with the time point of LPA production at 3 h, because cPLA_2_ and iPLA_2_ mediate PC hydrolysis into LPC, the precursor of LPA [35-37]. It should be noted that activated cPLA_2_ slowly reduced to the basal level from 3 h, while iPLA_2_ activity rapidly decreased from 2 h. The difference might be due to the distinct mechanisms of cPLA_2_ and iPLA_2_ activation, since cPLA_2_ can be activated by enhanced level of intracellular calcium, as seen in the calcium influx through NMDA receptor and calcium mobilization from endoplasmic reticulum mediated by NK1 receptor, while calcium-independent iPLA_2_ lacks the multiple triggers [18]. Moreover, it was interesting that MK-801, the antagonist of NMDA receptor, completely reversed the increased cPLA_2_ activity, but partially inhibited iPLA_2_ activity, which might be explained by the fact that the activity of cPLA_2_, but not iPLA_2_, is calcium-dependent. Furthermore, as BEL is a specific inhibitor of iPLA_2_ with high potency (half-maximal inhibitory concentration: 60 nM) [38], in this study, it even decreased the elevated iPLA_2_ activity to the level lower than the basal one. In addition, pharmacological blockade of cPLA_2_ or iPLA_2_ abolished nerve injury-induced cPLA_2_ and iPLA_2_ activations as well as LPA production.

Usually, saturated fatty acyl chains (16:0 and 18:0) are located in *sn*-1 position in phospholipids, while unsaturated ones (18:1) are in *sn*-2 position. However, there is a report that several PC molecules possess 18:1-chains in both *sn*-1 and *sn*-2 positions, such as diacyl-18:1/22:6, diacyl-18:1/20:4, diacyl-16:0/18:1 and diacyl-18:0/18:1, in the spinal cord [41]. In this study, we found there was no significant change in PLA_1_ activity after nerve injury. Therefore, it is suggested that the production of 18:1 LPA isoform is mainly generated by the action of PLA_2_, but not PLA_1_, and 18:1-fatty acyl chain is located in *sn*-1 position.

On the other hand, in this study, minocycline-induced blockade of microglial activation at early phase significantly inhibited nerve injury-induced LPA production and increased PLA_2_ activations, which confirmed the evidence that microglia plays important roles in LPA production. Indeed, previous study showed that both nerve injury and i.t. LPA injection induced phosphorylation of microglial p38 kinase, subsequent up-regulation of microglial activation-related gene and morphological change from ramified to amoeboid type [30].

Although the biomarker of activated iPLA_2_ is not available so far, we performed immunohistochemistry study to evaluate the cell-type expressing p-cPLA_2_ (activated cPLA_2_). It should be noted that p-cPLA_2_ was predominantly expressed in most of spinal neurons, with minor ones in microglia. The neuron-colocalized p-cPLA_2_ seemed to diffuse in slightly broader regions (mainly laminae I-IV) of spinal dorsal horn. This broader distribution was similar to the case with activated microglia after the nerve injury [30]. Moreover, since most of p-cPLA_2_-expressing neurons were observed in broader regions of dorsal horn, but not in line-up regions at superficial layers (lamina I-II), we speculated that the neurons expressing p-cPLA_2_ might be the interneurons in vicinity of microglia as well as second order neurons receiving pain transmission from primary afferent neurons. Considering that iPLA_2_ also predominantly expresses in neurons [42,43], and LPA can be synthesized and secreted by primary cultured neurons *in vitro*[44], we can hypothesize that spinal neurons, especially second order neurons and interneurons, are likely the cells responsible for the release of LPC/LPA, and the machineries may include the microglial activation.

It should be also noted that nerve injury-induced LPA production and increased PLA_2_ activities were completely absent in *Lpar1*^*-/-*^ and *Lpar3*^*-/-*^ mice, suggesting both LPA_1_ and LPA_3_ receptors were responsible for LPA synthesis, being consistent with the findings that both *Lpar1*^*-/-*^ and *Lpar3*^*-/-*^ mice abolished neuropathic pain behavior in response to LPA injection or nerve injury [20,29]. On the other hand, our RT-PCR results and other reports demonstrated that both LPA_1_ and LPA_3_ receptors expressed in microglia [14,45], while their levels in neurons were reported to be limited [13,46], indicating that microglial LPA_1_ and LPA_3_ receptors might induce the release of biological factors, which in turn activated cPLA_2_ or iPLA_2_ in neurons, leading to an LPA production.

We found that both 18:1 and 20:4 LPA preferentially activated LPA_1_ and LPA_3_ receptors, while 16:0, 18:0 and 14:0 LPA were poor agonists, being consistent with previous reports [47]. Since the level of 20:4 LPA in the spinal dorsal horn was under detection limit, even after nerve injury in the present MALDI-TOFMS system, it was evident that 18:1 LPA is the most functionally potent LPA molecule, which was produced after nerve injury. On the other hand, we found that only 18:1 LPA, but not 16:0 or 18:0 LPA, produced new LPA with three species (18:1, 16:0 and 18:0 LPA) at 1-3 h, among which 18:1 LPA was also the predominant product. This result was consistent with the behavior finding that only 18:1 LPA, but not 16:0 or 18:0 LPA, induced neuropathic pain-like behavior. Given the fact that 18:1 LPA was the most potent molecule to interact with both LPA_1_ and LPA_3_ receptors, we can conclude that 18:1 LPA plays major roles in LPA_1_ and LPA_3_ receptors-mediated amplification of LPA production, possibly through microglial activation.

## Conclusion

The present study demonstrates that 18:1 LPA is the major species of LPA in quantity and function in terms of LPA-induced amplification of LPA production. The mechanisms underlying the LPA production include the pain transmission by Glu and SP as well as indirect microglial activation, possibly through LPA_1_ and LPA_3_ receptors. The activation of cPLA_2_, which plays a key role for the production of LPC or LPA, is identified to be in neurons. Thus, produced LPA may work for the self-amplification via neuron-glia network. Targeted inhibition of 18:1 LPA or related pathways may be the potent strategy for the prevention of nerve injury-induced neuropathic pain.

## Methods and materials

### Animals

Male C57BL/6 J mice (TEXAM corporation, Nagasaki, Japan), homozygous mutant mice for the LPA_1_[48] and LPA_3_[49] receptor genes (*Lpar1*^*-/-*^ and *Lpar3*^*-/-*^), and their sibling wild-type mice from the same genetic background were used in this experiment. The subjects weighed 20-24 g. They were kept in a room maintained at 21 ± 2°C and 55 ± 5% relative humidity with a 12 h light/dark cycle, and had free access to a standard laboratory diet and tap water. The procedures were approved by the Nagasaki University Animal Care Committee, and complied with the fundamental guidelines for the proper conduct of animal experiments and related activities in academic research institutions under the jurisdiction of the Ministry of Education, Culture, Sports, Science and Technology, Japan.

### Drugs

18:1 LPA, MK-801 and minocycline were purchased from Sigma-Aldrich Co. (St. Louis, MO). 16:0, 17:0 and 18:0 LPA were purchased from Doosan Serdary Research Laboratories (Toronto, Canada). Monoisotopic ^68^Zn^2+^-Phos-tag (Phos-tag^®^) was obtained from the NARD Institute Ltd. (Hyogo, Japan) and MANAC Incorporated group (Hiroshima, Japan). CP-99994 was generously provided by Pfizer Pharmaceuticals (Sandwich, Kent, UK). AACOCF3 and BEL were purchased from Cayman Chemicals (Ann Arbor, MI).

For mass spectrometry experiments, various LPA species were dissolved in methanol (Sigma, St. Louis, MO). For *in vivo* experiments, all pharmacological antagonists except for minocycline were dissolved in artificial cerebrospinal fluid (125 mM NaCl, 3.8 mM KCl, 1.2 mM KH_2_PO_4_, 26 mM NaHCO_3_, 10 mM glucose). Minocycline was dissolved in physiological saline.

### Partial sciatic nerve ligation

Partial ligation of the sciatic nerves was performed under anesthesia with pentobarbital (50 mg/kg, i.p.), according to modified methods [6,30]. The common sciatic nerve of the right hind limb was exposed at the high thigh level through a small incision and the dorsal half of the nerve thickness was tightly ligated with a silk suture.

### Extraction of LPA from tissues

The unilateral dorsal half including dorsal horn (laminae I-V) of the lumber (L4-L6) spinal cord was removed. The averaged wet weight of the isolated unilateral spinal cord in each mouse was approximately 6.15 mg tissue weight. LPA were extracted from tissues according to modified methods [7,34]. After their isolation, the tissue samples were homogenized in 200 μl cold saline containing 100 mM of o-vanadate and 1 mM of EDTA. The homogenates were transferred into a glass tube (13 × 100 mm), and mixed with 0.5 nmol of 17:0 LPA, an internal standard, and 1 ml acetone. After vigorous vortex and centrifugation at 1300 × g for 5 min, the supernatant was discarded. The remaining pellet was washed twice with 0.5 ml acetone again, and dried with N_2_ gas. The dried pellet was mixed with 0.1 ml chloroform, 0.2 ml methanol and 0.08 ml water. After centrifugation at 1300 × g for 5 min, the supernatant was collected, and mixed with 0.2 ml chloroform, 0.2 ml 5% potassium chloride potassium chloride and 0.001 ml 28% aqueous ammonia. Following centrifugation at 1300 × g for 5 min, the supernatant was collected and washed with 0.4 ml chloroform/methanol (17:3, v/v). After washing for four times, 10 nmol of monoisotopic ^68^Zn^2+^-Phos-tag and 0.4 ml chloroform/methanol (17:3, v/v) were added to the supernatant (water/methanol phase). After shaking and centrifugation, the lower chloroform phase was collected, and the remaining water/methanol phase was extracted again. The combined chloroform phases were dried with N_2_ gas. The final sample was dissolved in 50 μl methanol containing 0.1% aqueous ammonia and stored at -20°C until use for analysis.

### MALDI-TOFMS analyses

One μl from 50 μl of finally obtained methanol solution was spotted on an MALDI plate (Bruker Daltonics, Inc., CA, USA). Immediately, 1 μl of THAP solution (10 mg/ml in acetonitrile; Sigma, St. Louis, MO) was layered on the mixture as matrix solution. After drying, the sample was applied to an Ultraflex^TM^ TOF/TOF systems (Bruker Daltonics, Inc., CA, USA). Mass spectrometry was performed in the positive mode, using an accelerating voltage of 25 kV. The laser energy was used at energy of 30 – 70% (3.0 – 7.0 μJ) and a repetition rate of 10 Hz. The mass spectra were calibrated externally using Peptide calibration standard (Bruker Daltonics, Inc., CA, USA) as a standard peptide calibration. Each spectrum was produced by accumulating data of 1500 or 2500 consecutive laser shots.

### Primary culture of mouse microglia

Glial cultures were prepared from the whole brain tissues of 1-day-old C57BL/6 J mice and maintained for 8–14 days in Dulbecco’s modified Eagle’s medium (DMEM) containing 10% fetal bovine serum (Gibco, Carlsbad, CA, USA), 1% penicillin/streptomycin (final concentration 100 units/ml; Invitrogen, Tokyo, Japan). All medium was changed every three days, from 24 h after the start of culture. The microglia was obtained as floating cells over the mixed glial culture, and seeded onto 6-well plate with the density of 1.0 × 10^5^ cells/cm^2^. After incubation at 37°C in a 5% CO_2_ atmosphere overnight, we removed the medium and added 400 μl TRIzol (Invitrogen, Carlsbad, CA, USA) into each well for RT-PCR experiment.

### RT-PCR

The expression levels of LPA receptors were evaluated by RT-PCR, according to described method [29,50]. Cultured microglia and isolated L4-6 SC from naive mice were lysed with TRIzol for RNA preparation. Total RNA (1 μg/sample) was used for cDNA synthesis with PrimeScript^®^ RT reagent Kit (Takara, Otsu, Japan). The cycling conditions for all primers were 3 min at 95°C, then 50 cycles of 30 s at 95°C, 30 s at 55°C and 2 min at 72°C. The PCR primer sequences were as follows: LPA_1_, 5’-ATCTTTGGCTATGTTCGCCA-3’ (forward) and 5’-TTGCTGTGAACTCCAGCCA-3’ (reverse); and for LPA_3_, 5’-TTGCCTCTGCAACATCTCGG-3’ (forward) and 5’-CATGACGGAGTTGAGCAGTG-3’ (reverse). Then, the PCR products were analyzed by 1.5% agarose gel electrophoresis.

### Thermal paw withdrawal test

In this test, nociception was measured as the latency to paw withdrawal evoked by exposure to a thermal stimulus [6,30,51,52]. Unanesthetized animals were placed in Plexiglas cages on top of a glass sheet and allowed an adaptation period of 1 h. A thermal stimulator (IITC Inc., Woodland Hills, CA) was positioned under the glass sheet and the focus of the projection bulb was aimed precisely at the middle of the plantar surface of the animal. A mirror attached to the stimulator permitted visualization of the plantar surface. A cut-off time of 20 s was set to prevent tissue damage.

### Phospholipase A_2_ activity assays

The activities of cPLA_2_ and iPLA_2_ were detected using the following assays as described previously [6,30,53]. Briefly, ipsilateral side of spinal dorsal horn was removed. After sonication and centrifugation at 20000 × *g* for 20 min at 4°C, the supernatant was collected and kept on ice. The protein concentration of the supernatant was determined by the Lowry method, and the assays were performed using a cPLA_2_ assay kit (Cayman Chemicals, Ann Arbor, MI, USA) to evaluate the cPLA_2_ activity or a modified cPLA_2_ assay kit (Cayman Chemicals, Ann Arbor, MI, USA) to evaluate the iPLA_2_ activity, as described previously [53]. In the cPLA_2_ assay, the tissue samples were incubated with both BEL, an iPLA_2_ inhibitor [38], and a substrate, arachidonoyl thio-PC, at 20°C for 1 h in a assay buffer. The reactions were stopped by DTNB/EGTA for 5 min, and the absorbances were determined at 405 nm using a standard plate reader. To detect the activity of iPLA_2_, but not cPLA_2_, the samples were incubated with the substrate, arachidonoyl thio-PC, at 20°C for 1 h in a modified Ca^2+^-free buffer [4 mM EGTA, 160 mM HEPES pH 7.4, 300 mM NaCl, 8 mM Triton X-100, 60% glycerol, 2 mg/ml of bovine serum albumin (BSA)]. The reactions were stopped by the addition of 5,5’-dithiobis(nitrobenzoic acid) for 5 min. The activity of PLA_2_ was defined as the percentage of the control activity as follows: injured tissues (absorbance/mg of protein)/normal tissues (absorbance/mg of protein) × 100.

### Phospholipase A_1_ activity assay

PLA_1_ activity assay was performed according to the manufacturer’s protocol of EnzChek^®^ phospholipase A1 assay kit (Invitrogen, Molecular Probes, OR, USA). Briefly, isolated ipsilateral spinal dorsal horn was sonicated in PLA_1_ reaction buffer (50 mM Tris-HCl, 0.14 M NaCl, 2 mM CaCl_2_, pH 7.4) and centrifuged at 20000 × *g* for 20 min at 4°C. The supernatant was collected and its protein concentration was determined by the Lowry method. In the PLA_1_ assay, the substrate-liposome mix was prepared by mixing 9 μl of lipid mix [3 μl of dioleoylphosphatidylcholine (10 mM; Sigma-Aldrich, St. Louis, MO), 3 μl of dioleoylphosphatidylglycerol (10 mM; Sigma-Aldrich, St. Louis, MO) and 3 μl of PLA_1_ substrate (1 mM PED-A1; Invitrogen, Molecular Probes, OR, USA)] and 501 μl of PLA_1_ reaction buffer. Subsequently, 1 μl of tissue sample, standard PLA_1_ (Sigma-Aldrich, St. Louis, MO) or negative control (PLA_1_ reaction buffer) was incubated with 19 μl of substrate-liposome mix in a 384-well microplate (Greiner bio-one, Frickenhausen, Germany) for 30 min. Then the fluorescence was measured using a PHERAstar FS reader (BMG-Labtech, Offenburg, Germany) equipped for excitation at 485 nm and fluorescence emission at 520 nm. The activity of PLA_1_ was defined as the percentage of the control activity as follows: injured tissues (absorbance/mg of protein) / normal tissues (absorbance/mg of protein) × 100.

### Immunohistochemistry

Mice were deeply anesthetized with pentobarbital (50 mg/kg) and perfused with potassium-free phosphate buffered saline (pH7.4), followed by 4% paraformaldehyde solution. L4-6 spinal cord segments were then isolated, postfixed in the same fixative for 3 h, and replaced with 25% sucrose overnight. Tissues were fast-frozen in cryo-embedding compound and cut on a cryostat at a thickness of 10 μm.

Immunofluorescence labeling was performed by blocking the sections with 2% BSA in TBST (0.1% Triton X-100 in Tris Buffered Saline) for 2 h at 20°C, followed by incubation with anti-NeuN antibody (1:500; anti-mouse, Chemicon, CA) or anti-Iba1 antibody (1:250; anti-goat, Abcam, Cambridge, UK) overnight at 4°C. After washing, sections were incubated with Alexa Fluor 594-conjugated anti-mouse IgG (1:300; Invitrogen, Carlsbad, CA) or Alexa Fluor 546-conjugated anti-goat IgG (1:300; Invitrogen, Carlsbad, CA), respectively, for 2 h at 20°C. Then, sections were blocked with 2% BSA in TBST for 2 h at 20°C and incubated overnight at 4°C with anti-phospho-cPLA_2_ antibody (anti-rabbit, 1:100; Abcam, Cambridge, UK). Sections were subsequently incubated with secondary antibody, Alexa Fluor 488-conjugated anti-rabbit IgG (1:300; Invitrogen, Carlsbad, CA), for 2 h at 20°C. Stained sections were washed and cover-slipped with Perma Fluor (Thermo Shandon, Pittsburgh, PA, USA). Images were collected using an LSM 710 confocal microscope with ZEN software (Carl Zeiss, Oberkochen, Germany).

### Calcium mobilization assay

LPA_1_-B103 or LPA_3_-B103 cells were used for calcium mobilization assay. The cells were harvested by centrifugation and re-suspended with 0.1% BSA supplied-DMEM. The cell suspension was plated 30 μl per well in a 384-well plate with the density of 5.0 × 10^3^ cells/well. Following incubation at 37°C in a 5% CO_2_ atmosphere overnight, cells were loaded with 10 μl Fluo-8 (8 μM) in 0.1% BSA supplied-DMEM containing 1 mg/ml amaranthl. After 30 min, 20 μl various LPA species at defined concentration was added, and fluorescence was recorded by Functional Drug Screening System/μCell (Hamamatsu Photonics K.K., Hamamatsu city, Japan) immediately. The fluorescence intensity was described as signal ratio (tested value / basal value).

### Statistical analysis

Statistical analysis was evaluated using the Tukey’s multiple comparison *post hoc* analysis following one-way ANOVA. The criterion of significance was set at *p* < 0.05. All results are expressed as mean ± SEM.

## Abbreviations

LPA: Lysophosphatidic acid; i.t.: Intrathecal; Lpar1-/- or Lpar3-/-: *Lpar1*- or *Lpar3*-deficient mice; LPC: Lysophosphatidylcholine; MALDI-TOFMS: Matrix-assisted laser desorption/ionization time-of-flight mass spectrometry; Glu: Glutamate; SP: Substance P; NK1: Neurokinin 1; cPLA2: Cytosolic phospholipase A_2_; iPLA2: Calcium-independent phospholipase A_2_; PC: Phosphatidylcholine; AACOCF3: Arachidonyl trifluoromethyl ketone; BEL: Bromoenol lactone; i.p.: Intraperitoneal; PLA1: Phospholipase A_1_; RT-PCR: Reverse transcription polymerase chain reaction; NeuN: Neuronal nuclei; Iba1: Ionized calcium-binding adaptor molecule 1; p-cPLA2: Phosphorylated cPLA_2_; LPA1-B103 or LPA3-B103: B103 cells expressing LPA_1_ or LPA_3_ receptor; ED50: Half-maximal effective dose; DMEM: Dulbecco’s modified Eagle’s medium; BSA: bovine serum albumin; TBST: 0.1% Triton X-100 in Tris Buffered Saline.

## Competing interests

The authors declare that they have no competing interests.

## Authors’ contributions

LM was responsible for experimental design, performance of all the experiments except for calcium mobilization assay, and writing the manuscript. JN conducted MALDI-TOFMS analyses and performed calcium mobilization assay as well as PLA_1_ activity assay. JC generated *Lpar1*^*-/-*^ and *Lpar3*^*-/-*^ mice. HU was responsible for experimental design and manuscript revision for publication. All authors read and approved the final manuscript.
